# Interprofessional recognition of audiology scope of practice in hearing and balance care: Evidence from the University of Sharjah

**DOI:** 10.1371/journal.pone.0347133

**Published:** 2026-04-09

**Authors:** Muhammed Ayas, Rohit Ravi, Dhanshree R. Gunjawate

**Affiliations:** 1 Audiology & Speech Language Pathology, College of Health Sciences, University of Sharjah, Sharjah, United Arab Emirates; 2 Department of Audiology & Speech Language Pathology, Kasturba Medical College Mangalore, Manipal Academy of Higher Education, Manipal, India; LSU Health Shreveport, UNITED STATES OF AMERICA

## Abstract

**Objective:**

Clear recognition of professional scope of practice (SOP) supports timely referral and effective interprofessional care in hearing and balance services. While audiology’s role in hearing assessment is well recognised, understanding of its broader scope, particularly in vestibular care during professional training, remains unclear.

**Methods:**

A descriptive cross-sectional online questionnaire was administered to health-sciences students and faculty. Scenario-based classification tasks assessed attribution of audiology SOP across hearing-loss diagnosis, balance-disorder care, and a multidisciplinary vignette involving hearing loss with dizziness. A composite SOP recognition outcome was derived and logistic regression identified predictors of full SOP recognition.

**Results:**

A total of 235 participants were analysed (95.7% students). Recognition of audiology’s role in hearing-loss diagnosis was high (90.0%). Attribution in balance-disorder care was lower (73.0%) and more variable. Audiology was included in the multidisciplinary vignette by 85.6% of respondents. Full SOP recognition was observed in 71.2%. Higher audiology awareness was independently associated with full SOP recognition (OR 1.80; 95% CI 1.34–2.42).

**Conclusions:**

While audiology’s role in hearing care is widely recognised, scope attribution remains variable in vestibular and multidisciplinary contexts. Given the predominance of pre-licensure students in the sample, findings should be interpreted as reflecting professional role conceptualisation during training rather than established clinical practice. These findings highlight the importance of explicit role clarification within health-sciences education to support consistent and effective interprofessional engagement across hearing and balance care pathways.

## Introduction

Audiologists play a central role in the assessment and management of hearing and balance disorders. Their scope of practice (SOP) includes diagnostic evaluation, rehabilitation, long-term management, and patient education across the lifespan, frequently delivered within multidisciplinary healthcare settings [1,2]. As healthcare systems increasingly emphasise integrated, patient-centred models of care, clarity regarding professional SOP has become essential for effective interprofessional collaboration [3]. Within multidisciplinary teams, optimal hearing and balance care depends on clear delineation of clinical responsibilities across audiology, otolaryngology, neurology, physiotherapy (PT), and other allied health professions.

SOP clarity is particularly important for clinical presentations involving overlapping symptom profiles, such as dizziness and combined auditory-vestibular complaints, where diagnostic and management pathways may span multiple disciplines. Interprofessional literature suggests that audiology’s contribution to holistic hearing and balance care is not always consistently recognised unless explicitly articulated within collaborative or educational frameworks [4,5]. Evidence from vestibular and hearing care pathways further demonstrates that referral patterns and service delivery models vary across clinical contexts, often reflecting differences in role visibility and system integration rather than professional capability [6,7]. These observations indicate that the understanding and operationalisation of audiology SOP may vary according to training exposure and professional context.

From a health-systems perspective, accurate SOP attribution underpins efficient referral pathways, reduces duplication of services and supports timely access to appropriate assessment and intervention. When professional roles are clearly understood, patients are more likely to enter appropriate care pathways earlier in their clinical journey. Conversely, ambiguity in role attribution, particularly for complex presentations involving concurrent hearing and balance symptoms, may contribute to fragmented care trajectories, delayed intervention, and inefficiencies in service delivery and workforce utilisation [1,3,8]. Despite its importance, empirical evidence examining how clinical responsibilities are attributed across disciplines remains limited.

Much of the existing literature has focused on general awareness of audiology or perceptions of interprofessional collaboration, rather than explicitly assessing scenario-based attribution of diagnostic and management responsibilities for hearing and balance disorders [4,5,9]. Few studies have examined whether SOP recognition is consistent across differing clinical contexts, such as hearing loss alone, balance disorders, and combined auditory-vestibular presentations. Moreover, structured attempts to quantify comprehensive SOP recognition using classification-based approaches remain scarce. As professional roles continue to evolve within increasingly complex health systems, understanding how SOP attribution develops during health-sciences education represents a critical yet underexplored area [10].

Examining SOP attribution during pre-licensure education and within academic health-sciences environments is particularly relevant, as professional role perceptions formed during training may influence subsequent clinical decision-making, referral behaviours, and collaborative practice. Clear role delineation has been associated with improved interprofessional coordination and collaborative readiness, whereas ambiguity may contribute to uncertainty when managing complex clinical presentations [10,11]. Importantly, recognised audiology SOP frameworks include both hearing and vestibular assessment and management within audiological practice [1,2]. However, the extent to which these defined roles are consistently recognised across health-sciences disciplines during training remains unclear. Addressing this gap is increasingly relevant as healthcare systems adopt integrated care models that rely on accurate role attribution to optimise workforce utilisation and patient outcomes.

To address this gap, the present study examines attribution of audiology clinical SOP for hearing and balance care among health-sciences students and faculty. The study aims to: (i) assess attribution of hearing-loss diagnosis to audiology; (ii) examine recognition of audiology’s role in balance disorder assessment and management; (iii) evaluate professional inclusion patterns within a multidisciplinary clinical vignette involving concurrent hearing loss and dizziness; and (iv) identify predictors of comprehensive SOP recognition, including audiology awareness, prior exposure, and disciplinary background.

## Materials and methods

### Study design

This study employed a descriptive cross-sectional design conducted within a multidisciplinary College of Health Sciences at the University of Sharjah in the United Arab Emirates. The study was conducted in accordance with the principles of the Declaration of Helsinki and received ethical approval from the University of Sharjah Research Ethics Committee (REC-24-11-18-01-F). Electronic informed consent was obtained from all participants prior to participation.

### Participants

Undergraduate and postgraduate students, as well as faculty members across health-sciences disciplines, were invited to participate via institutional email distribution and classroom-based recruitment methods (including in-class QR code access). Students and faculty were included to capture SOP attribution within a health-sciences educational environment, reflecting perspectives during professional formation as well as among educators involved in training. The survey was distributed across programmes within the College of Health Sciences using broad institutional dissemination rather than a defined sampling list of confirmed recipients. Consequently, the exact number of individuals who viewed or received the invitation could not be determined, and a precise response rate could not be calculated. The data were collected using a structured online questionnaire administered between January 2025 and October 2025. Participation was voluntary and anonymous. A total of 243 individuals completed the survey. Respondents who provided data for at least one SOP attribution item were included in the analysis, yielding a final analytic sample of 235 participants. The study size was determined pragmatically based on the total number of eligible students and faculty available during the data-collection period. To minimise potential response and social desirability bias, responses were self-administered and participation was independent of any academic evaluation.

### Instrument and measures

The questionnaire was adapted from previously published instruments examining audiology awareness and interprofessional role clarity [12], with additional items developed specifically to assess discipline-based attribution of hearing and balance responsibilities. Newly created items underwent internal review by three audiology professionals to ensure content relevance, conceptual alignment, and clarity of wording. The role-attribution component of the questionnaire was designed as a scenario-based classification task rather than a psychometric scale. Accordingly, items were intended to assess the accuracy with which respondents attributed clinical responsibilities to professional disciplines, rather than their attitudes, beliefs, or perceptions toward audiology. Traditional psychometric validation metrics were not applicable to this component.

The final instrument comprised four domains and was administered in English: (i) demographic characteristics; (ii) awareness of audiology; (iii) previous exposure and interprofessional collaboration; and (iv) clinical role-attribution scenarios (S1 file). SOP recognition was measured using three multi-response items:

a) Hearing-loss attribution: selection of professions responsible for diagnosing hearing loss.
b) Balance-disorder attribution: selection of professions involved in diagnosing or managing balance disorders.
c) Multidisciplinary case vignette: selection of appropriate professionals for managing a patient with concurrent hearing loss and dizziness.

These scenarios were selected to reflect core clinical pathways in hearing and vestibular care, progressing from single-domain presentations (hearing loss alone; balance disorders alone) to a combined auditory-vestibular presentation requiring multidisciplinary involvement. This design allowed examination of whether SOP attribution was consistent across increasingly complex and realistic clinical contexts.

### Outcome and Predictor variables

Responses were recoded into dichotomous variables indicating whether audiology was selected in each attribution context. Audiology was classified as a correct attribution in each scenario based on internationally recognised SOP frameworks for audiology in hearing and vestibular care [1,2]. A composite SOP recognition score (range 0–3) was generated by summing correct attributions across the three scenarios. Participants selecting audiology in all scenarios were classified as demonstrating full SOP recognition. Dichotomisation was applied to represent a conceptually meaningful threshold of comprehensive role recognition across clinical contexts, rather than partial or context-specific attribution.

Predictor variables were selected a priori based on theoretical relevance and existing literature on interprofessional role clarity. These included age group, academic role (student vs faculty), and discipline group. For analytic consistency, disciplines were categorised as follows: PT, Nursing (NSG), Clinical Nutrition (CN), Medical Laboratory Sciences (MLS), Environmental Health Sciences (EHS), Medical Diagnostic Imaging (MDI), Health Care Management (HCM), Audiology & Speech-Language Pathology (ASLP), and Other (OTH). The Other category comprised participants from smaller health-sciences programmes not represented in sufficient numbers for independent analysis (public health, biotechnology, molecular medicine disciplines). Participants from the ASLP discipline were retained in the analysis to allow comparison of attribution patterns across disciplines and served as the reference category in regression analyses. Awareness of audiology was assessed using a five-point Likert-type item (1 = not aware at all to 5 = extremely aware). Previous exposure to audiology was coded dichotomously (yes/no) based on reported encounters with audiology-related discussions or clinical scenarios during training.

### Statistical analysis

Descriptive statistics were used to summarise participant characteristics and scope-of-practice attribution outcomes. Categorical variables were reported as frequencies and percentages. Associations between participant characteristics and scope-of-practice attribution were examined using chi-square tests. A multivariable logistic regression model was used to identify predictors of full scope-of-practice recognition, with adjusted odds ratios (ORs) and 95% confidence intervals (CIs) reported. ASLP discipline served as the reference category. Statistical significance was set at *p* < 0.05. Analyses were performed using complete-case data; respondents with missing data for a given analysis were excluded pairwise.

## Results

### Participant characteristics

Of the 243 individuals who completed the questionnaire, 235 provided data for at least one SOP role-attribution item and were included in the analysis. Most respondents were students (95.7%). Accordingly, findings primarily reflect SOP recognition during professional training rather than established clinical practice. Participants were predominantly aged ≤20 years (66.4%) or 21–24 years (24.7%). Disciplines represented included PT (23.8%), NSG (20.9%), ASLP (17.4%), MDI (8.9%), CN (9.4%), MLS (7.7%), EHS (5.1%), HCM (3.4%) and OTH (3.4%). The mean audiology awareness score was 3.26 (SD 1.41), and 32.3% reported previous exposure to audiology. Participant characteristics are presented in Table 1.

**Table 1:** Participant characteristics (n = 235).

Variable	Category	n	%
Age	20 and below	156	66.4
	21-24	58	24.7
	25-29	5	2.1
	30-34	5	2.1
	35-39	2	0.9
	40 and above	9	3.8
Role	Student	225	95.7
	Faculty member	10	4.3
Year in programme	1st year	118	52.4
	2nd year	50	22.2
	3rd year	31	13.8
	4th year	26	11.6
Discipline	Audiology & Speech-Language Pathology	41	17.4
	Clinical Nutrition	22	9.4
	Environmental Health Sciences	12	5.1
	Health Care Management	8	3.4
	Medical Diagnostic Imaging	21	8.9
	Medical Laboratory Sciences	18	7.7
	Nursing	49	20.9
	Other*	8	3.4
	Physiotherapy	56	23.8
Previous exposure to audiology	No	159	67.7
	Yes	76	32.3
Audiology awareness score	Mean (SD)	3.26	1.41

* OTH = participants from smaller or heterogeneous health-sciences disciplines (public health, biotechnology, molecular medicine).

### Recognition of audiology in hearing-loss attribution

Overall, 90.0% of respondents identified audiology as a profession responsible for diagnosing hearing loss. Recognition was high across most disciplines, with ASLP and HCM demonstrating complete recognition (100%). PT, NSG, CN, EHS, MLS and MDI groups reported high recognition levels (range 89–95%). The OTH group reported the lowest recognition (53%).

### Recognition of audiology in balance-disorder attribution

Across the sample, 73.0% of respondents selected audiology as being involved in the diagnosis and management of balance disorders. Recognition was highest among ASLP respondents (95.0%), followed by PT (80.0%). Lower proportions were observed in NSG (65.0%), EHS (67.0%), MLS (56.0%), and the OTH group (35.0%). Across all respondents, PT was selected by 60.0% and otolaryngology (ENT) by 9.0% as being involved in balance-disorder care. This finding should be interpreted in the context of the item wording, which focused on diagnostic and management responsibility rather than specialist referral pathways.

### Multidisciplinary professional inclusion patterns

**Fig 1:**
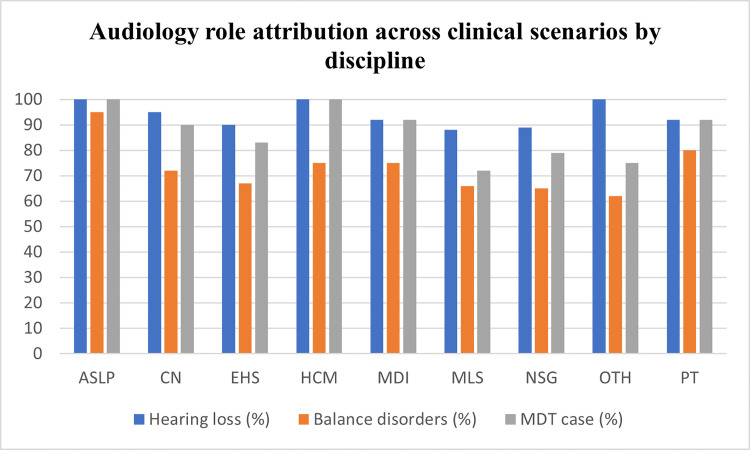
Audiology role attribution across clinical scenarios by discipline. ASLP = Audiology & Speech-Language Pathology; PT = Physiotherapy; NSG = Nursing; CN = Clinical Nutrition; MLS = Medical Laboratory Sciences; MDI = Medical Diagnostic Imaging; EHS = Environmental Health Sciences; HCM = Health Care Management; OTH = Other. *Grouped bar chart showing the proportion of respondents in each discipline who selected Audiology as responsible for the diagnosis of hearing loss, the diagnosis and/or management of balance disorders, and inclusion in multidisciplinary care for concurrent hearing loss and dizziness.*

**Fig 2:**
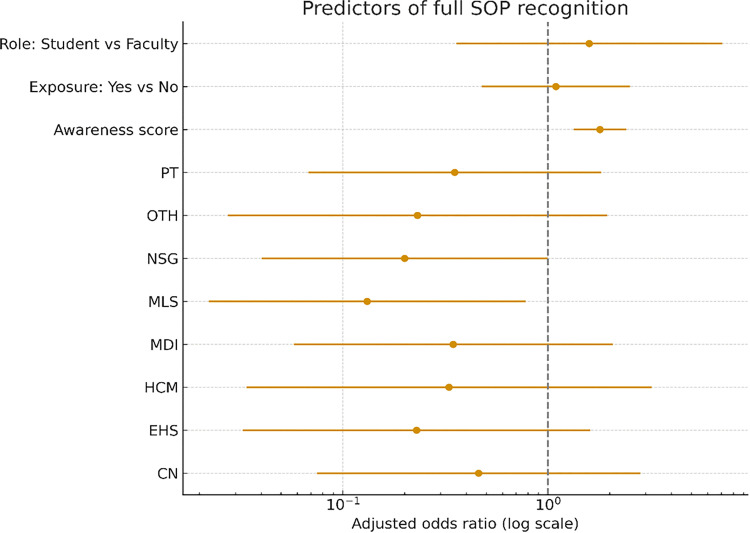
Audiology scope-of-practice recognition across increasing clinical complexity. Line graph illustrating the proportion of respondents selecting audiology across three clinical contexts: hearing-loss diagnosis, balance-disorder care and a multidisciplinary vignette involving concurrent hearing loss and dizziness. Data are shown separately for ASLP and non-ASLP disciplines to illustrate changes in SOP recognition with increasing clinical complexity.

In the vignette involving hearing loss with dizziness, 85.6% of respondents included an audiologist in the multidisciplinary care team. Otolaryngology (ENT) was selected by 68.7% of respondents, PT by 45.7% and general practice by 44.4%. Selection of otolaryngology (ENT) without audiology involvement was observed in 4.9% of participants. Percentages within the OTH discipline group are reported descriptively and should be interpreted cautiously due to the heterogeneous composition and smaller sample size of this category. Discipline-level patterns across the three clinical scenarios are illustrated in Fig 1. When the three clinical contexts are considered sequentially, a reduction in audiology role attribution with increasing clinical complexity is observed, particularly among non-ASLP disciplines (Fig 2).

### Composite SOP recognition

The composite SOP recognition score (range 0–3) had a mean of 2.49 (SD 0.93). Full SOP recognition (score = 3) was achieved by 71.2% (167/235) of respondents. SOP recognition differed significantly across disciplines (χ² (8) = 32.9, p < 0.001). The highest proportions of full recognition were observed among ASLP (95.1%), PT (80.4%), HCM (75.0%), CN (71.4%) and MDI (71.4%). Lower proportions were recorded in EHS (58.3%), MLS (55.6%), NSG (63.3%) and OTH (29.4%). Among student respondents, full SOP recognition was not associated with year of study (χ²(3) = 0.03, p = 0.99), suggesting consistent attribution patterns across training stages.

### Predictors of full SOP recognition

**Fig 3:**
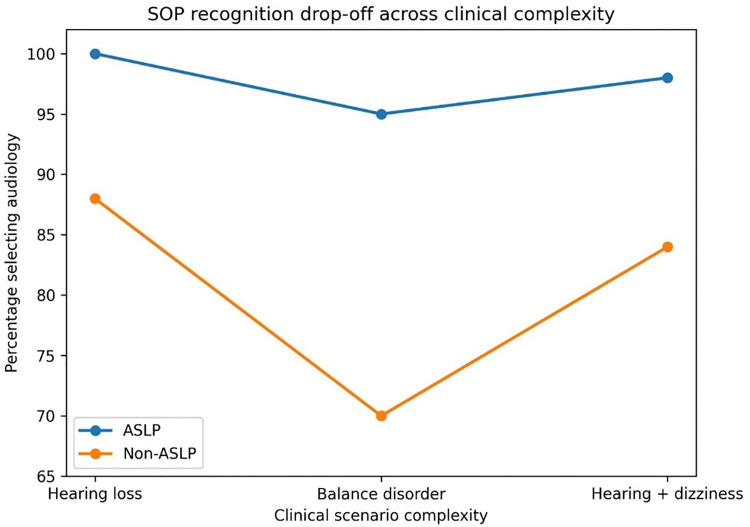
Predictors of full recognition of audiology SOP. Forest plot showing adjusted odds ratios (ORs) and 95% confidence intervals from a multivariable logistic regression model with full SOP recognition as the outcome. ASLP served as the reference discipline (not shown). Exact confidence intervals are reported in the text. Abbreviations as in Fig 1. The model additionally includes audiology awareness score, prior exposure to audiology (Yes vs No), and academic role (Student vs Faculty).

Higher audiology awareness scores were independently associated with increased odds of full SOP recognition (OR 1.80; 95% CI 1.34–2.42; p < 0.001). Compared with ASLP, MLS participants had significantly lower odds of achieving full SOP recognition (OR 0.13; 95% CI 0.02–0.78; p = 0.026). NSG participants also demonstrated lower odds, at the threshold of statistical significance (OR 0.20; 95% CI 0.04–1.00; p = 0.050). Previous exposure to audiology and academic role (student vs faculty) were not significantly associated with full SOP recognition. Regression results are presented in Fig 3.

## Discussion

This study provides a cross-disciplinary examination of how health-sciences students and faculty attribute the audiologist’s SOP in relation to hearing and balance care within a multidisciplinary academic setting. Three key findings emerged. First, recognition of audiology’s role in hearing-loss diagnosis was high across most disciplines. Second, attribution of balance-disorder assessment and management to audiology was lower and showed greater variability across disciplines. Third, although most respondents included audiology in a multidisciplinary scenario involving concurrent hearing loss and dizziness, a small proportion demonstrated single-profession attribution in this context. Taken together, these findings identify areas of both consistency and variation in SOP attribution that are relevant to health-sciences education and collaborative care models. These findings should therefore be interpreted as reflecting SOP recognition during professional training rather than representing established clinical practice among working professionals.

### Recognition of audiology in hearing-loss diagnosis

Attribution of hearing-loss diagnosis to audiology was high across nearly all disciplines, consistent with previous reports indicating that hearing assessment is the most widely recognised component of audiological practice [12–14]. This pattern likely reflects the long-standing integration of audiometry within clinical care pathways and the growing visibility of hearing-related services across healthcare settings [1,14]. The comparatively lower recognition observed within the OTH group suggests that familiarity with hearing-care pathways may differ across academic programmes, underscoring the importance of incorporating foundational knowledge of hearing-health roles across a broad range of health-sciences curricula.

### Attribution of audiology’s role in balance-disorder care

Recognition of audiology’s involvement in balance-disorder diagnosis and management was lower than for hearing loss and varied more substantially across disciplines. This pattern aligns with existing literature indicating that vestibular assessment remains less consistently attributed to audiology outside disciplines with direct exposure to balance science and rehabilitation [15,16]. The observed variation across NSG, MLS, EHS, and OTH respondents likely reflects differences in how vestibular roles are emphasised or encountered during training, rather than professional exclusion from balance care. Evidence from studies of dizzy patient pathways further highlights the need for clearer role delineation within multidisciplinary care models [15]. Given the prevalence of dizziness and imbalance as presenting complaints, improved clarity regarding professional roles in vestibular assessment may support more consistent and timely multidisciplinary engagement [6]. In this context, audiology’s expanding diagnostic role in vestibular assessment is increasingly recognised. Inconsistent scope recognition during training may therefore translate into delayed or fragmented vestibular care pathways in practice.

### Single-profession attribution in multidisciplinary clinical scenarios

In the multidisciplinary vignette describing concurrent hearing loss and dizziness, most respondents appropriately included audiology as part of the care team. A small proportion selected otolaryngology without audiology involvement. Although infrequent, this pattern suggests that simplified role conceptualisations may persist in complex clinical scenarios involving overlapping auditory and vestibular symptoms. Similar pathway simplifications have been described in the literature on dizziness and vertigo care and are often attributed to the complexity of clinical presentation rather than intentional professional exclusion [17]. Evidence from multidisciplinary vestibular care models further emphasises the importance of reinforcing complementary, rather than exclusive, professional roles to support coordinated assessment and management [18]. Importantly, the low prevalence of single-profession attribution observed in this study indicates that most respondents recognised the relevance of audiology within shared-care contexts.

### Why SOP recognition matters for health systems and practice

Accurate SOP recognition has practical implications for healthcare delivery beyond professional awareness. Clear role attribution supports more efficient referral pathways by directing patients to appropriate diagnostic services earlier in their clinical journey. This is particularly important for balance disorders where delayed vestibular assessment is common [6,16,17]. Improved SOP recognition may reduce duplication of investigations, limit unnecessary specialist referrals, and optimise utilisation of audiology and allied health services [8,11]. In the context of vestibular care, earlier recognition of audiology’s diagnostic role may facilitate timely vestibular testing, support targeted rehabilitation, and reduce prolonged symptom burden for patients [15,18]. From a workforce perspective, clearer SOP attribution contributes to more effective task distribution within multidisciplinary teams. This alignment of professional expertise with clinical need supports integrated, patient-centred models of care [10,19].

### Audiology awareness as a predictor of SOP recognition

Audiology awareness emerged as the strongest predictor of full SOP recognition across hearing-loss, balance-disorder, and multidisciplinary scenarios. Higher awareness scores were associated with a greater likelihood of accurately attributing audiology’s role across all contexts. In contrast, prior exposure to audiology was not independently associated with full SOP recognition after accounting for awareness. This suggests that exposure alone may not translate into accurate role understanding unless accompanied by explicit emphasis on professional scope [20,21]. These findings are consistent with educational literature demonstrating that structured and intentionally designed learning experiences are more effective in shaping professional role understanding than incidental or observational exposure alone [22,23].

### Educational and workforce considerations

From an educational perspective, variability in SOP attribution across disciplines highlights opportunities for targeted curricular enhancement. Integrating vestibular science and scenario-based interprofessional learning into health-sciences curricula may support more consistent role conceptualisation during professional formation. Explicit discussion of professional boundaries and complementarities during training has been associated with improved collaborative readiness and role clarity within the health workforce [21,24,25]. Importantly, strengthening SOP understanding during education may have downstream effects on clinical practice. This may influence referral behaviour, interprofessional communication, and care coordination as graduates transition into the workforce [25–27]. While this study did not examine clinical outcomes directly, examining SOP attribution during training provides a relevant foundation for future research. Future studies may link educational interventions to service delivery and patient outcomes [28]. Scenario-based classification tasks, such as those used in this study, have been increasingly applied in health-professions education research to assess applied role understanding in clinically realistic contexts. These findings may inform understanding of how SOP recognition develops during professional training across health-sciences education contexts.

### Limitations

This study examined how professional roles are conceptualised during health-sciences training rather than observed clinical decision-making or referral behaviour. Attribution of audiology SOP was assessed using self-reported responses rather than direct observation of practice, and faculty participation was limited relative to student representation. Although year of study was examined, the cross-sectional design limits inference regarding how SOP recognition evolves longitudinally across training. In addition, inclusion of ASLP participants, who demonstrated expectedly high SOP recognition, may have contributed to higher overall recognition estimates within the sample. The questionnaire focused on core hearing and balance responsibilities and did not examine other domains of audiology practice, such as tinnitus management or hearing-device provision. The single‑institution nature of this study necessitates cautious interpretation of the findings, which may not extend to other universities or countries with differing health‑sciences training frameworks. Despite these limitations, the study provides a structured and clinically grounded examination of SOP recognition across hearing, balance, and multidisciplinary clinical contexts, offering insights relevant to interprofessional education and service pathway design.

## Conclusion

This study examined attribution of audiology SOP for hearing and balance care among health-sciences students and faculty within a multidisciplinary academic setting. While recognition of audiology’s role in hearing-loss diagnosis was high across disciplines, attribution of audiology’s role in balance-disorder care and in multidisciplinary clinical scenarios demonstrated greater variability. Audiology awareness emerged as the strongest predictor of comprehensive SOP recognition, underscoring the relevance of explicit role clarification during professional training. These findings support the need for further research examining how SOP recognition during health-sciences education translates to interprofessional practice and clinical care pathways.

## Supporting information

S1 File Questionnaire. (PDF)

S2 File Dataset. (XLSX)
