# Supplementary material for: Interprofessional recognition of audiology scope of practice in hearing and balance care: Evidence from the University of Sharjah
Source: PLoS One. 2026 Apr 9;21(4):e0347133. doi: 10.1371/journal.pone.0347133 (PMC13065007; doi:10.1371/journal.pone.0347133)
Supplement: S1 File — (PDF) [file pone.0347133.s001.pdf]

Section 1: Demographic Information

1. **Age:**

    - 20 and below
    - 21-24
    - 25-29
    - 30-34
    - 35-39
    - 40 and above

2. **Role at the University:**

    - Student (*Skip to question 4)*
    - Faculty member *(Skip to question 8)*

(For Students Only)

3. **Current Educational Level:**

    - Undergraduate
    - Postgraduate
    - Doctoral

4. **Year in Program:**

    - 1st Year
    - 2nd Year
    - 3rd Year
    - 4th Year
    - Other (please specify)

5. **Major:**

    - Physiotherapy
    - Nursing
    - Medical Diagnostic Imaging
    - Clinical Nutrition
    - Health Care Management
    - Medical Laboratory Sciences
    - Environmental Health Sciences
    - Other (please specify)

6. **Are you currently enrolled in a clinical practicum in your major?**

    - Yes
    - No

(For Faculty Only)

8. **Professional Background:**

    - Physiotherapy
    - Nursing
    - Medical Diagnostic Imaging
    - Clinical Nutrition
    - Health Care Management
    - Medical Laboratory Sciences
    - Environmental Health Sciences
    - Other (please specify)

9. **Years in Profession:**

    - Less than 1 year
    - 2-5 years
    - 6-10 years
    - 11-15 years
    - 16-20 years
    - 21+ years

Section 2: Scope of Practice Awareness

10. **How aware are you of the role of audiologists in general healthcare?**

    - Not aware at all
    - Slightly aware
    - Moderately aware
    - Very aware
    - Extremely aware

11. **In your educational or professional experience, have you encountered any discussions or situations involving audiology?**

    - Yes
    - No
    - If yes: Please describe the context or situation.

12. **Which profession(s) can diagnose hearing loss?** (Select all that apply)

- Audiology
- Physiotherapy
- Nursing
- Medical Diagnostic Imaging
- Clinical Nutrition
- Health Care Management
- Medical Laboratory Sciences
- Environmental Health Sciences
- Other (please specify)

13. **Which profession(s) do you associate with the diagnosis and management of balance disorders?** (Select all that apply)

- Audiology
- Physiotherapy
- Nursing
- Medical Diagnostic Imaging
- Clinical Nutrition
- Health Care Management
- Medical Laboratory Sciences
- Environmental Health Sciences
- Other (please specify)

14. **A 50-year-old patient presents with hearing loss and dizziness. Given this presentation, which healthcare professionals would be most appropriate to collaborate in a multidisciplinary approach for treatment?** (Select all that apply)

- Audiologist
- Physiotherapist
- Ear Nose Throat Specialist
- General Practitioner
- Other (please specify)

Section 3: Interprofessional Education and Collaboration

15. **Have you participated in any interprofessional education (IPE) activities that included audiology?**

- Yes
- No

- If yes: Please specify the activity

16. **To what extent do you believe interprofessional education (IPE) enhances collaboration between audiologists and other healthcare professionals?**

    - Not at all
    - A little
    - Somewhat
    - Quite a bit
    - A great deal

17. **In your opinion, how important is it for audiologists to be involved in interprofessional teams?**

    - Not important at all
    - Slightly important
    - Moderately important
    - Very important
    - Extremely important

18. **How often do you collaborate with audiologists or other professionals in your practice or studies?**

    - Never
    - Rarely
    - Sometimes
    - Often
    - Always

19. **Rate the effectiveness of your collaboration with audiologists or other professionals:**

    - Very ineffective
    - Ineffective
    - Neutral
    - Effective
    - Very effective

Section 4: Knowledge and Interaction with Professions

20.How much do you know about the following professions?
(Select the level that best describes your knowledge)

Profession	Not at all	A little	Somewhat	Quite a bit	A lot/This is my profession
Audiology	○	○	○	○	○
Speech-Language Pathology	○	○	○	○	○
Physiotherapy	○	○	○	○	○
Nursing	○	○	○	○	○
Medical Diagnostic Imaging	○	○	○	○	○
Clinical Nutrition	○	○	○	○	○
Health Care Management	○	○	○	○	○
Medical Laboratory Sciences	○	○	○	○	○
Environmental Health Sciences	○	○	○	○	○

21. Have you directly interacted with professionals in the following fields?

Profession	Yes	No
Audiology	○	○
Speech-Language Pathology	○	○
Physiotherapy	○	○
Nursing	○	○
Medical Diagnostic Imaging	○	○
Clinical Nutrition	○	○
Health Care Management	○	○
Medical Laboratory Sciences	○	○
Environmental Health Sciences	○	○

22. How likely are you to refer a patient to the following professions?

Profession	Very unlikely	Unlikely	Neutral	Likely	Very likely
Audiology	○	○	○	○	○
Speech-Language Pathology	○	○	○	○	○
Physiotherapy	○	○	○	○	○
Nursing	○	○	○	○	○
Medical Diagnostic Imaging	○	○	○	○	○
Clinical Nutrition	○	○	○	○	○
Health Care Management	○	○	○	○	○

Medical Laboratory Sciences	○	○	○	○	○
Environmental Health Sciences	○	○	○	○	○

23.How important do you perceive the role and scope of audiology as a healthcare profession?

- Not important at all
- Slightly important
- Neutral
- Very important
- Extremely important
